# Lumped Plasticity Model and Hysteretic Performance of Ultra-High-Performance Concrete Rocking Pier

**DOI:** 10.3390/ma16196515

**Published:** 2023-09-30

**Authors:** Haifang He, Yulong Zhou, Shoushan Cheng, Hongyi Liu

**Affiliations:** National Engineering Laboratory of Bridge Safety and Technology (Beijing), Research Institute of Highway Ministry of Transport, Beijing 100088, China; hf.he@rioh.cn (H.H.); ss.cheng@rioh.cn (S.C.); hongyi.liu@ctvic.cn (H.L.)

**Keywords:** rocking pier, ultra-high-performance concrete, lumped plasticity model, hysteretic performance, parametric analysis

## Abstract

Rocking piers using ultra-high-performance concrete (UHPC) have high damage-control capacity and self-centering characteristics that can limit the post-earthquake recovery time of bridges. To study the hysteretic behavior of UHPC rocking piers, a lumped plasticity model is proposed that comprises two parallel rotational springs and which can accurately calculate their force-displacement hysteretic behavior. Three states of the rocking piers, decompression, yield, and large deformation, are considered in this study. The model is verified based on existing experimental results, and the hysteretic characteristics of the UHPC rocking piers, such as strength, stiffness, and energy dissipation, are further analyzed. The research results show that the lumped plasticity analysis model proposed in this study can predict the force-displacement hysteretic behavior of the rocking piers accurately. Moreover, the hysteretic performance of the UHPC rocking piers is better than that of rocking piers using normal-strength concrete. An increase in the energy dissipation reinforcement ratio, pre-stressed tendon ratio, and initial pre-stress improves the lateral stiffness and strength of the UHPC rocking piers. However, the increase in the pre-stressed tendon ratio and initial pre-stress reduces their energy-dissipation capacity.

## 1. Introduction

Numerous post-earthquake investigations have shown that the traffic interruption caused by earthquake damage to bridges significantly affects the post-disaster rescue and reconstruction operations in the stricken areas. This results in indirect losses that are difficult to estimate and far exceed social expectations [1,2]. Therefore, an important concept in the future seismic design of bridge structures is to effectively control earthquake damage and shorten the recovery time after an earthquake. Currently, the ductile seismic design method is primarily used in the seismic design of bridges in China; it experiences serious plastic damage and large residual displacements during earthquakes, which is highly unfavorable to the recovery of bridge capacity after earthquakes. Therefore, in recent times, a rocking structure with strong earthquake recovery ability has been widely focused on as a new seismic system for bridge structures.

**Figure 1:**
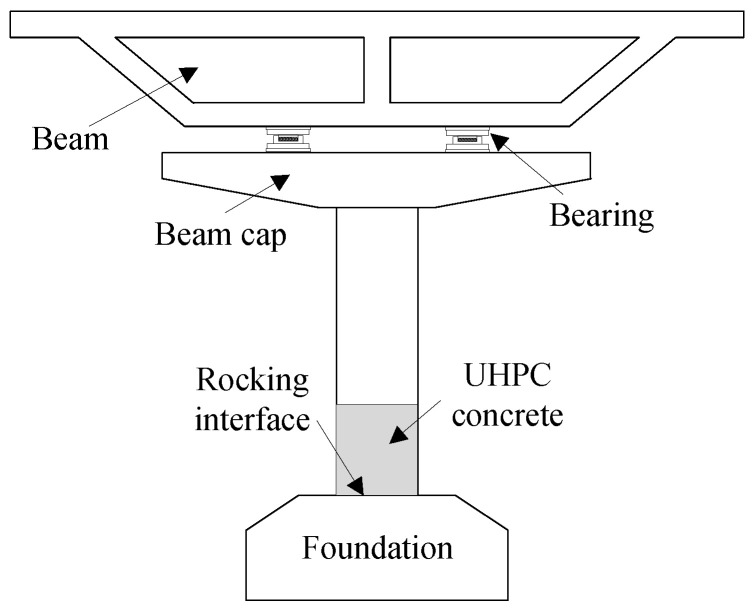
Schematic diagram of UHPC rocking pier.

Recently, the seismic concept of rocking bridges has been applied to bridge engineering [3,4,5,6,7]. In 1963, Housner [8] proposed for the first time the seismic response of a structure that was weakened by the rocking of available structures during earthquakes and presented a model to analyze the rocking behavior of rigid bodies. Subsequently, the Housner rigid-body rocking model was improved, and its applicability became possible [9,10,11,12,13]. Mander et al. [14] conducted a quasi-static experimental study on a rocking pier with only unbonded pre-stressed steel bars and proposed a simplified calculation formula for its force–displacement relationship. Pampanin et al. [15] and Palermo et al. [16] proposed the combined application of built-in energy-dissipating reinforcements and unbonded pre-stressed technology to improve the energy-dissipation capacity of a rocking pier. Guo et al. [17,18] adopted fiber-reinforced materials and modified alloys to improve the durability of pre-stressed tendons and energy-dissipating devices in corrosive environments. At present, seismic studies on rocking piers are still in the stage of structural research and basic theoretical development. Although the advantages of the seismic toughness of rocking piers have been confirmed via experiments and numerical methods, the hysteretic and post-seismic resilience of rocking piers using new materials, such as ultra-high-performance concrete (UHPC), remain to be further studied. UHPC is a reasonable combination of an ultra-fine admixture, ultra-high-strength steel fibers, an efficient water-reducing agent, and small particle aggregates [19,20]. Compared with ordinary concrete, UHPC has the characteristics of high strength, ductility, and durability. The seismic damage of a rocking pier is mainly the compressive damage of the column toe (concrete at the edge of the rocking interface). The application of UHPC to the plastic hinge area of a rocking pier (as shown in Figure 1) can significantly improve the compressive bearing capacity of its column toe, which allows for the limitation of seismic damage and failure of the bridge pier to a greater degree. At present, the multi-axial spring model is widely used to simulate the hysteretic behavior of rocking piers, but it is complex to determine the stiffness of the multi-axial springs. Therefore, a simple and clear analysis model of a UHPC rocking pier needs to be developed.

In this study, a UHPC rocking pier is taken as the research object, and the relationship between the height of the compression zone on the rocking interface and the stress states of the pre-stressed tendon and energy-dissipating steel bar are carefully considered. The section analysis of the rocking pier is divided into the analyses of self-centering and energy-dissipating components, and then a double-plastic hinge analysis model of the rocking pier is established. The hysteretic properties of the UHPC rocking bridge pier are analyzed. The influence of the energy dissipation reinforcement ratio, pre-stressed reinforcement ratio, and initial pre-stress ratio on the strength, stiffness, and energy-dissipation characteristics of UHPC rocking bridge piers is investigated. The obtained results are compared with those of a normal rocking bridge analysis. Based on the above, the research findings can provide reference for the seismic design of bridge structures and foundations.

## 2. Double-Plastic Hinge Analysis Model

### 2.1. Mechanical Mechanism

**Figure 2:**
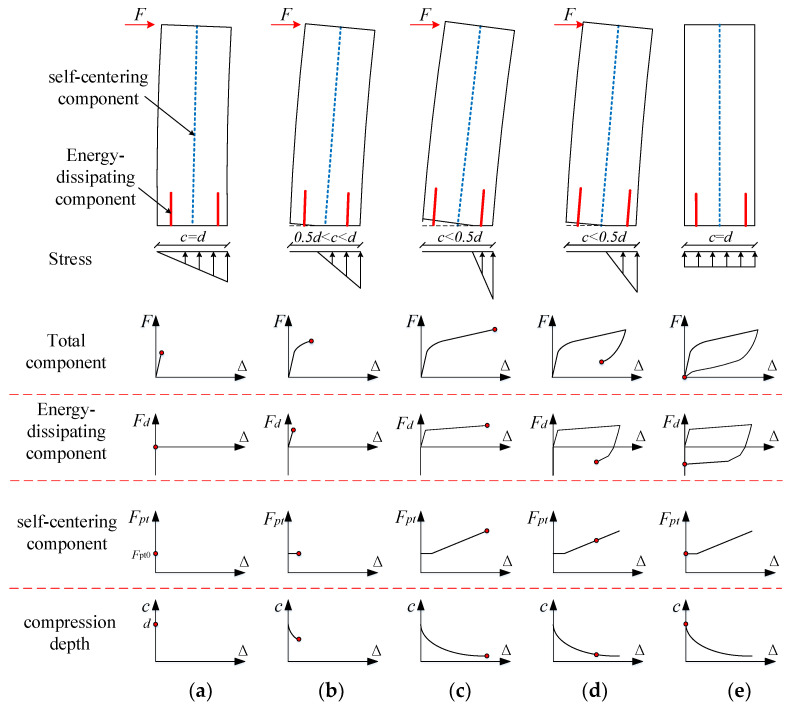
Deformation mode of rocking pier (*c* is the compression depth, *d* is the width of the column, *θ* is the rotation of the rocking interface. *F* and ∆ are the lateral force and displacement of the rocking pier, respectively. *F_d_* and *F_pt_* are the lateral forces of the energy-dissipating component and self-centering component, respectively. *F_pt_*_0_ is the initial pre-stress force.). (**a**) Decompression stage, (**b**) Yield stage, (**c**) Large deformation stage, (**d**,**e**) Re-centering stage.

A rocking bridge pier is placed directly on the top of a bearing platform to form a rocking interface, and the bridge pier and the bearing platform are connected as a whole by an unbonded pre-stressed tendon and an energy-dissipating device. As shown in Figure 2, under the action of an earthquake, the rocking pier, as the main compression member, is lifted and closed on the cap. Therefore, the plastic deformation of the pier is concentrated on the rocking interface, avoiding damage to the pier body. The rocking of the pier causes the unbonded pre-stressed tendon and the energy-dissipating device to provide the functions of self-centering and dissipation of seismic energy, respectively. The bending moments applied by the energy-dissipating and self-centering components and the height of the compression zone on the rocking interface change with the change in the rotation angle of the rocking interface during the deformation of the rocking pier. The bending moment–rotation and height of the compression zone in a typical deformation process are shown in Figure 2.

The stress stage of the rocking pier can be divided into four parts. In the first stage, named the decompression stage, as shown in Figure 2a, the stress of the concrete at the edge of the rocking pier is zero. In the second stage, named the yield stage, as shown in Figure 2b, openings appear at the rocking interface, and the assumption of a flat section is not satisfied. Therefore, introducing a displacement coordination condition to conduct a moment-angle analysis of the rocking interface is necessary [21]. In the third stage, named the large deformation stage, as shown in Figure 2c, the displacement coordination condition of the rocking pier is similar to that of the equivalent concept of the yield state. In the fourth stage, named the reset stage, as shown in Figure 2d, the lateral displacement of the rocking pier and the angle of the rocking interface decrease until the elastic displacement of the rocking pier and the angle of the rocking interface are zero (as shown in Figure 2e).

### 2.2. Analytical Model

Based on the force mechanism described in the above section, the moment-rotation relationship of a rocking pier is decomposed into a self-centering component composed of superstructure gravity and a pre-stress tendon and an energy-dissipating component composed of an energy-dissipating reinforcement. Considering the pressure release, yield, and large deformation of a rocking pier, the compressive strains at the outermost edge of the rocking interface in the three stress states are calculated.

(1) $$\varepsilon_{c}=\left\{ \begin{array}{l} \frac{2\left(N+F_{pt,0}\right)}{E_{c}BD}\;\;\;\;\;\;\;\;\;\;\;\;\;\;\;\;\;\;\;\;\;\;\;\;\;\;\;\;\;\;\;\theta =0\\ \left(\frac{3\theta }{l_{cant}}+\varphi_{dec}\right)\cdot c\;\;\;\;\;\;\;\;\;\;\;\;\;\;\;\;\;\;\;\;\;0<\theta \leq \frac{1}{3}\left(\varphi_{y}-\varphi_{dec}\right)l_{cant}\\ \left[\frac{\frac{3\theta }{l_{cant}}-\left(\varphi_{y}-\varphi_{dec}\right)}{\frac{3l_{p}}{l_{cant}}\left(1-\frac{l_{p}}{2l_{cant}}\right)}+\varphi_{y}\right]\cdot c\;\;\;\;\;\;\;\theta >\frac{1}{3}\left(\varphi_{y}-\varphi_{dec}\right)l_{cant} \end{array}\right.,$$

where *N* is the superstructure gravity, *F_pt_*_,0_ is the initial tension of the pre-stressed tendon, *E_c_* is the elastic modulus of concrete, *B* is the width of the rocking interface, *D* is the height of the rocking interface, *θ* is the rotation angle of the rocking interface, *l_cant_* is the calculated height of the rocking pier, *φ_dec_* is the pressure relief limit state curvature of the rocking pier, *C* is the compression depth of the rocking pier, *Y* is the yield state curvature of the rocking pier, and *L_p_* is the equivalent plastic hinge length of the rocking pier.

The strain of the *i*th layer pre-stressed tendon (*ε_pt,i_*) can be calculated using the following formula:

(2) $$\varepsilon_{pt,i}=\varepsilon_{pt,0}+\frac{\theta \left(d_{pt,i}-c\right)}{l_{pt}}=\frac{F_{pt,0}}{E_{pt}\cdot A_{pt,i}}+\frac{\theta \left(d_{pt,i}-c\right)}{l_{pt}}$$

where *ε_pt,_*_0_ is the initial tensile strain of the pre-stressed tendon, *d_pt,i_* is the distance between the *i*th layer pre-stressed tendon and the edge of the compression zone on the rocking interface, *l_pt_* is the unbonded length of the pre-stressed tendon, *E_pt_* is the elastic modulus of the pre-stressed tendon, and *A_pt,i_* is the section area of the *i*th layer pre-stressed tendon.

If the energy-dissipating reinforcement strain of the rocking pier is lower than the yield strain, *ε_y_*, the energy-dissipating reinforcement strain of the *i*th layer (*ε_ms,i_*) can be calculated using the following formula:

(3) $$\varepsilon_{ms,i}=\frac{\theta_{imp}\left(d_{ms,i}-c\right)}{l_{ms}+\frac{4}{3}l_{sp}},$$

where *d_ms,i_* is the distance between the energy-dissipating device of the *i*th root and the edge of the compression zone of the rocking interface, *l_sp_* is the strain distribution length, and *l_ms_* is the unbonded length of the energy-dissipating reinforcement.

If the strain of the energy-dissipating steel bar exceeds the yield strain (*ε_y_*), its strain in the *i*th layer (*ε_ms,i_*) is calculated using the following formula:

(4) $$\varepsilon_{ms,i}=\frac{\theta_{imp}\left(d_{ms,i}-c\right)}{l_{ms}+2l_{sp}},$$

When the strain is defined on the entire interface, the stresses of the self-centering and energy-dissipating components can be calculated to satisfy the force equilibrium condition. The bending moment bearing capacity of the rocking interface is typically calculated according to the centroid of the concrete compression zone. In this process, the bending moments generated by the self-centering and energy-dissipating components of the rocking pier can be calculated.

**Figure 3:**
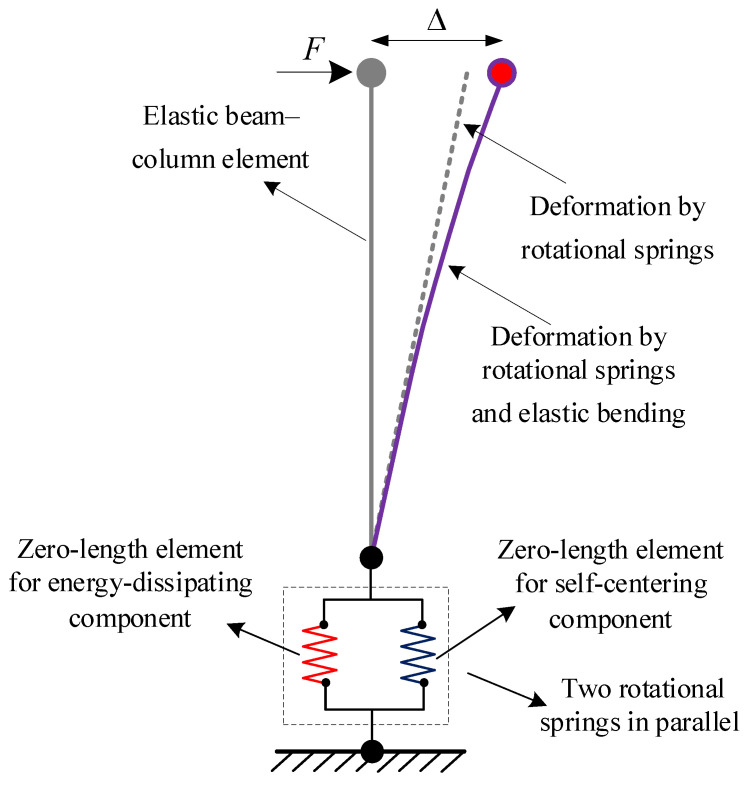
Lumped plasticity analysis model.

A lumped plasticity model as shown in Figure 3 is built using the OpenSees finite element analysis platform (Version 2.2.2). An elastic beam-column element is applied to simulate the lateral elastic stiffness of the pier. Two zero-length elements are used to simulate the moment-rotation hysteretic behaviors of the self-centering component and the energy-dissipating component, respectively. Then, the two zero-length elements are connected in parallel to the elastic beam-column element. The moment-rotation hysteretic behavior of the self-centering component is simulated using the ElasticMultiLinear material model. The moment-rotation hysteretic behavior of the energy-dissipating component is simulated using the RambergOsgoodSteel material model. Based on the mechanical mechanism, the moment-rotation hysteretic behaviors of the self-centering component and energy-dissipating component can be obtained using the deformation mode. In addition, the compression depth of the rocking pier and the rotation can be calculated using an iterative method proposed in the literature [21].

### 2.3. Model Verification

**Figure 4:**
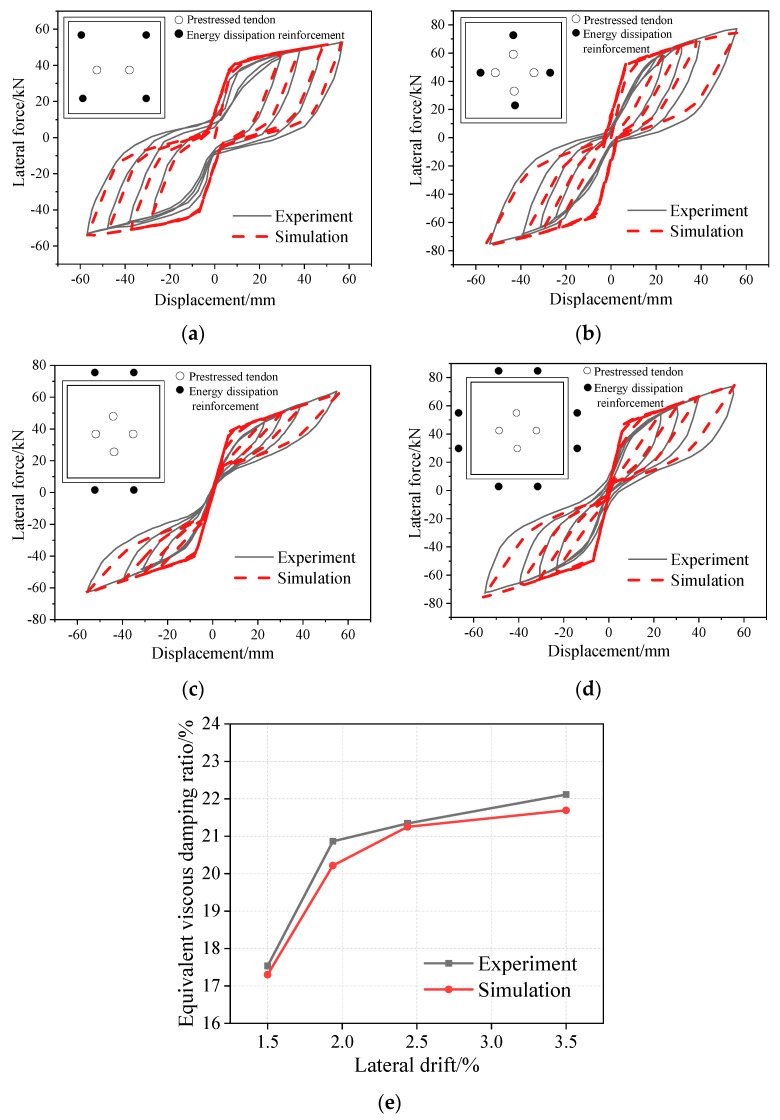
Comparisons between the experimental and simulated results of the hysteretic curves. (**a**) Hysteretic curves of HBD1, (**b**) Hysteretic curves of HBD2, (**c**) Hysteretic curves of HBD4, (**d**) Hysteretic curves of HBD5, (**e**) Equivalent viscous damping ratio of HBD1.

Based on the studies by Marriot et al. [21,22,23], 1:3-scaled quasi-static test results of a rocking pier are used to verify the validity of the double-plastic hinge analysis model. The calculated height of the test pier is 1.6 m, and the width of the rectangular section is 0.35 m. The section layouts of the selected rocking pier specimens—HBD1, HBD2, HBD4, and HBD5—are shown in Figure 4, in which the energy-consuming steel bars of HBD1 and HBD2 are internal, whereas those of HBD4 and HBD5 are external. The quasi-static loading regime is consistent with that used in the literature [21]. Also, the parameters used for the modeling of the rocking pier specimens HBD1, HBD2, HBD4, and HBD5 can be seen in the literature [21]. The force-displacement hysteretic curves of the four specimens are calculated using the double-plastic hinge analysis model proposed in the above section and compared with the test results. Based on the comparison shown in Figure 4, the double-plastic hinge analysis model can better simulate the hysteretic behavior of the rocking pier. Also, the equivalent viscous damping ratio is closer between the experimental and simulated results, as shown in Figure 4e. However, the analysis model overestimated the initial stiffness, due to the lack of protection of the pier rocking toe. Therefore, this analysis model is more suitable for a rocking pier with enough protection of the rocking toe. Also, the soil-structure interaction effects are not considered in this study; this is a worthy topic for future research [24].

## 3. Hysteretic Performance and Parameter Analysis of UHPC Rocking Bridge Pier

### 3.1. Hysteretic Properties of UHPC Rocking Bridge Pier

In this section, the hysteretic properties of a UHPC rocking bridge pier are compared with those of the pier specimen HBD2. The stress-strain model of UHPC adopts that of concrete proposed by Scott et al. [25]. Wang et al. [26] confirmed the accuracy of the model proposed by Scott et al. [25] applied to unconstrained and constrained UHPC. For constrained UHPC, the peak stress (*σ_con,pk_*), peak strain (*ε_con,_*_0_), limit stress (*σ_con,_*_20_), and limit strain (*ε_con,_*_20_) can be calculated as follows:

(5) $$\sigma_{con,pk}=\sigma_{un,pk}\left(1+1.8\rho_{w}f_{yv}/\sigma_{un,pk}\right),$$

(6) $$\varepsilon_{con,0}=\varepsilon_{un,0}\left(1+5\rho_{w}f_{yv}/\sigma_{un,pk}\right),$$

(7) $$\sigma_{con,20}=\lambda_{con,rs}\sigma_{con,pk},$$

(8) $$\varepsilon_{con,20}=0.06\left(1-\lambda_{con,rs}\right)+\lambda_{con,rs}\varepsilon_{con,0},$$

where *σ_un,pk_* is the unconstrained peak stress of UHPC, *ε_un,_*_0_ is the peak strain of unconstrained UHPC, *ρ_w_* is the volume reinforcement ratio of the stirrup, *f_yv_* is the yield stress of the stirrup, and *λ_con,rs_* is the strain coefficient of constrained UHPC, which is generally set at 0.2.

In this study, the concrete is replaced with UHPC based on the specimen HBD2, and then a UHPC rocking bridge pier is established. Wang et al. [26] showed that the compressive strength and elastic modulus of UHPC are 124.3 MPa and 48,000 MPa, respectively. A double-plastic hinge model of this UHPC rocking pier was established, the lateral cyclic load of the HBD2 rocking pier was applied to the double-plastic hinge model, and the corresponding hysteretic curve was obtained.

**Figure 5:**
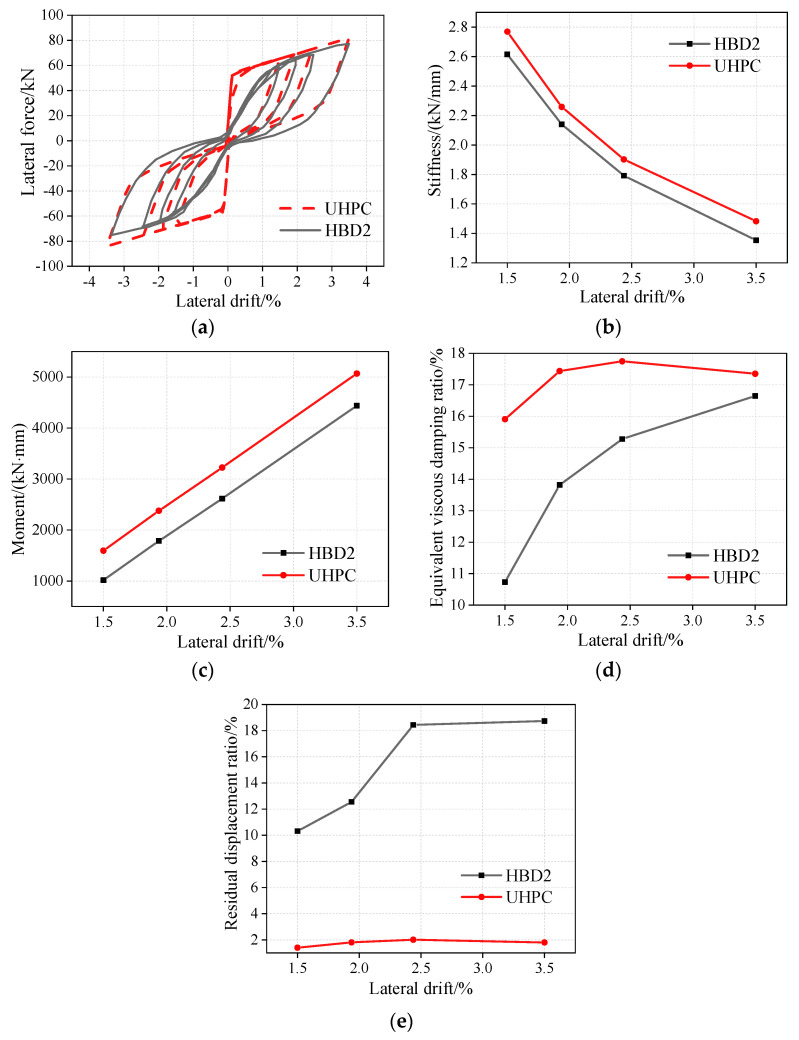
Hysteretic performance comparison of UHPC and HBD2 rocking piers. (**a**) Hysteretic curves, (**b**) Stiffness degradation curves, (**c**) Moment of energy-dissipating components, (**d**) Equivalent viscous damping ratio, (**e**) Residual displacement ratio.

As shown in Figure 5a, the UHPC rocking pier has larger initial stiffness and lateral bearing capacity than HBD2. As shown in Figure 5b, with an increasing drift rate, the secant stiffness change trends of the specimens HBD2 and UHPC are the same; however, the secant stiffness of specimen UHPC is higher than that of specimen HBD2. For example, under a drift rate of 1.5%, the secant stiffness of UHPC is 7.7% higher than that of HBD2 (2.6 kN/mm). At a drift rate of 3.5%, the secant stiffness of UHPC is 9.6% higher than that of HBD2.

As shown in Figure 5c, with an increase in the drift rate, the variation trends of the energy dissipation moments of HBD2 and UHPC are the same; however, the energy dissipation moment of UHPC is larger than that of HBD2. For example, at a drift rate of 1.5%, the energy dissipation torques of HBD2 and UHPC are 1016.6 kN·mm and 1594.8 kN·mm, respectively. Thus, the energy dissipation torque of UHPC is 56.9% larger than that of HBD2. At a drift rate of 2.4%, the energy dissipation moment of UHPC is 23.3% larger than that of HBD2.

As shown in Figure 5d, when the drift rate is lower than 3.5%, the initial stage of the equivalent viscous damping ratio of specimen UHPC is greater than that of specimen HBD2. Specifically, under a drift rate of 1.5%, the equivalent viscous damping ratios of specimens UHPC and HBD2 are 15.9% and 10.7%, respectively. At a drift rate of 3.5%, the corresponding values are 17.4% and 16.6%. The equivalent viscous damping, an index of energy-dissipation capacity, can be calculated using the following formula:

(9) $$\xi_{eq}=\Delta W_{i}/(2\pi ku_{\max}^{2})$$

where Δ*W_i_* is the dissipated energy in one hysteretic loop, *k* is the secant stiffness, and *u*_max_ is the maximum displacement in a cycle.

In this study, the residual displacement ratio is used to quantitatively examine the self-centering ability of the specimens. A large residual displacement ratio implies a poor self-centering ability. As shown in Figure 5e, under a drift rate of 2.4%, the residual displacement ratios of HBD2 and UHPC are 2.0% and 18.4%, respectively. Therefore, UHPC has a small residual displacement ratio and good self-centering ability.

### 3.2. Parameter Analysis of UHPC Rocking Pier

The strength, stiffness, and energy-dissipation characteristics of the UHPC rocking pier were studied using the developed double-plastic hinge analysis model. The parameters mainly included the reinforcement ratio of the energy-dissipating steel bar, initial pre-stress force, and reinforcement ratio of the pre-stress tendon.

#### 3.2.1. Reinforcement Ratio of the Energy-Dissipating Steel Bar

**Figure 6:**
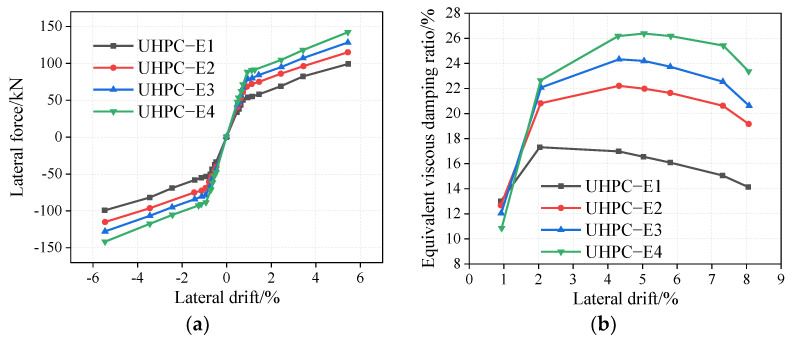
Influence of energy dissipation reinforcement ratio on UHPC rocking piers. (**a**) Envelop curves, (**b**) Equivalent viscous damping ratio.

The reinforcement diameters of the rocking bridge pier specimens UHPC-E1, UHPC-E2, UHPC-E3, and UHPC-E4 are 12.5 mm, 16 mm, 18 mm, and 20 mm, respectively. The corresponding steel reinforcement ratios are 0.40%, 0.66%, 0.83%, and 1.03%. As shown in Figure 6a, with an increase in the energy consumption steel reinforcement ratio, the UHPC rocking lateral bearing capacity of the bridge pier increases. For example, under a drift rate of 5.44%, as the energy consumption steel reinforcement ratio increases from 0.40% to 1.03%, the lateral force increases by 43.43%. Before yielding, the pier is in the elastic stage, the slope of the curve increases gradually, and the initial stiffness increases gradually. After yielding, the curve slope is basically the same; therefore, the stiffness of the UHPC rocking pier is basically the same. As shown in Figure 6b, with an increase in the steel reinforcement ratio of the energy-dissipating steel bar, under the same drift, the energy consumption and equivalent viscous damping ratio of the bridge pier are remarkably improved. For example, at a drift rate of 4.31%, the equivalent viscous damping ratio increases by 5.22% when the reinforcement ratio of the energy-dissipating bar increases from 0.40% to 0.66%.

#### 3.2.2. Initial Pre-Stress Force

**Figure 7:**
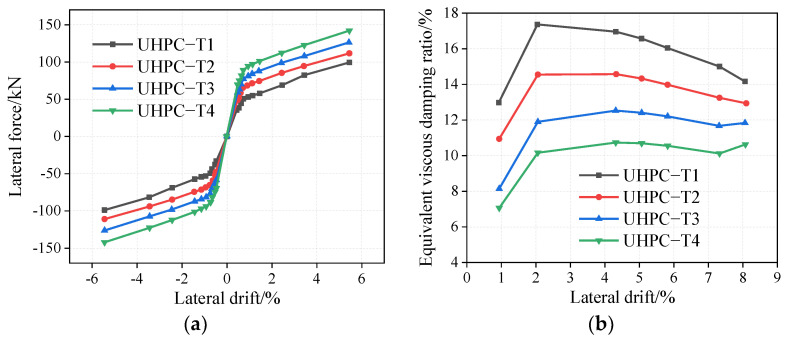
Influence of initial pre-stress on UHPC rocking piers. (**a**) Envelop curves, (**b**) Equivalent viscous damping ratio.

The initial pre-stress force of the tendons of the rocking pier specimens UHPC-T1, UHPC-T2, UHPC-T3, and UHPC-T4 are 75 kN, 112.5 kN, 150 kN, and 187.5 kN, respectively. As shown in Figure 7a, with an increase in the initial pre-stress force, the rocking UHPC lateral bearing capacity of the bridge pier increases. For example, under a drift rate of 5.44%, when the initial pre-stress force increases from 75 kN to 150 kN, the lateral force increases by 27.27%. Before yielding, the pier is in the elastic stage, and the slope of the curve increases gradually, indicating that the initial stiffness of the rocking pier can be improved by increasing the initial pre-stress. After yielding, the curve slope is basically the same; therefore, the stiffness of the UHPC rocking pier remains basically the same. As shown in Figure 7b, with an increase in the initial pre-stress force, under the same drift, the equivalent viscous damping ratio is significantly reduced due to the decrease of openings on the rocking interface. For example, at a drift rate of 4.31%, the equivalent viscous damping ratio decreases from 10.73% to 16.94% as the initial pre-stress force increases from 75 kN to 187.5 kN.

#### 3.2.3. Reinforcement Ratio of Pre-Stressed Tendon

**Figure 8:**
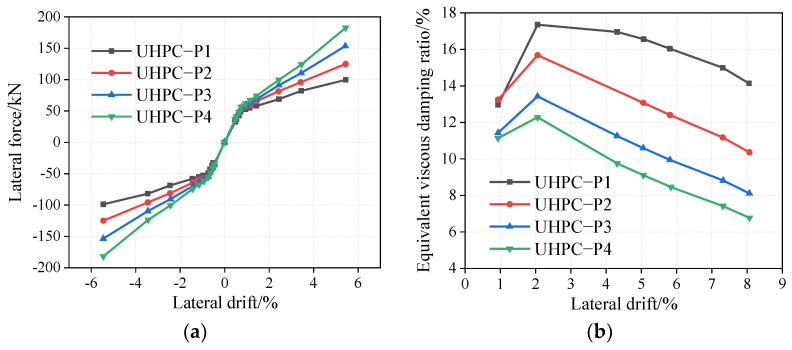
Influence of pre-stressed tendon ratio on UHPC rocking piers. (**a**) Envelop curves, (**b**) Equivalent viscous damping ratio.

The pre-stressed tendon areas of the rocking pier specimens UHPC-P1, UHPC-P2, UHPC-P3, and UHPC-P4 are 99 mm^2^, 198 mm^2^, 297 mm^2^, and 396 mm^2^, respectively. The corresponding ratios of the pre-stressed tendons are 0.32%, 0.65%, 0.97%, and 1.29%. As shown in Figure 8a, with an increase in the pre-stress reinforcement ratio, the lateral bearing capacity of the UHPC rocking bridge pier increases. For example, under a drift rate of 5.44%, when the pre-stressed tendon ratio increases from 0.32% to 1.29%, the lateral force increases by 83.84%. Before yielding, the bridge pier is in the elastic stage, and the curve slope and the initial stiffness are basically unchanged. The slope of the curve increases gradually after yielding; therefore, the stiffness of the UHPC rocking pier increases gradually after yielding. As shown in Figure 8b, with an increase in the pre-stress reinforcement ratio, under the same drift ratio, the equivalent viscous damping ratios of the UHPC rocking bridge piers are significantly reduced. For example, at a drift rate of 5.81%, the equivalent viscous damping ratio decreases from 16.02% to 8.46% when the reinforcement ratio increases from 0.32% to 1.29%.

## 4. Conclusions

In this study, a double-plastic hinge analysis model of a rocking pier is established based on its three stress states: compressive, yield, and large deformation. The hysteretic performance of a UHPC rocking pier is compared with that of an ordinary concrete rocking pier. The main conclusions of the parameter analyses of the UHPC rocking pier are as follows:

(1) The hysteretic behavior of the UHPC rocking bridge pier is studied based on the rocking bridge pier-loading mechanism. The dissipation yield pressures and large deformations of the three types of stress states of the rocking bridge pier are fully considered. Moreover, the bending moment–corner relations of the rocking bridge pier are divided into those of self-centering and energy-dissipating components. Accordingly, this paper proposes a double-plastic hinge analysis model that can finely calculate the force–displacement hysteretic behavior of the rocking bridge pier.
(2) The effectiveness of the model is verified using existing experimental results, and the hysteretic curves from the model simulation and experiments fit well. Moreover, the hysteretic properties, such as strength, stiffness, and energy dissipation of the UHPC rocking pier, are further analyzed.
(3) This study adopts the double-plastic hinge analysis model, which can accurately simulate the force–displacement hysteretic behavior of the rocking bridge pier. The rocking hysteretic performance of the UHPC rocking bridge piers is verified to be better than that of ordinary concrete rocking bridge piers. Moreover, compared to an ordinary concrete rocking bridge pier, the UHPC rocking bridge pier has higher lateral stiffness and strength and a higher energy-dissipation capacity and reset ability.
(4) The reinforcement ratio of the energy-dissipating reinforcement and an increase in the initial pre-stress can improve the lateral stiffness and strength of the UHPC rocking pier. For example, the lateral bearing capacity increases by approximately 30% when the initial pre-stress doubles. However, the equivalent viscous damping ratio decreases and the energy-dissipation capacity of the pier decreases with an increase in the reinforcement ratio of the pre-stress tendon and the initial pre-stress.

## Data Availability

Data sharing is not applicable to this article.
